# Macroecological predictors of evolutionary and plastic potential do not apply at microgeographic scales for a freshwater cladoceran under climate change

**DOI:** 10.1093/evlett/qrad042

**Published:** 2023-10-12

**Authors:** Christopher P Nadeau, Mark C Urban

**Affiliations:** Schoodic Institute at Acadia National Park, Winter Harbor, ME, United States; Ecology and Evolutionary Biology Department, University of Connecticut, Storrs, CT, United States; Center for Biological Risk, University of Connecticut, Storrs, CT, United States

**Keywords:** critical thermal maximum, *Daphnia*, evolution, freshwater rock pool, heritability, microgeographic adaptation

## Abstract

Rapid evolutionary adaptation could reduce the negative impacts of climate change if sufficient heritability of key traits exists under future climate conditions. Plastic responses to climate change could also reduce negative impacts. Understanding which populations are likely to respond via evolution or plasticity could therefore improve estimates of extinction risk. A large body of research suggests that the evolutionary and plastic potential of a population can be predicted by the degree of spatial and temporal climatic variation it experiences. However, we know little about the scale at which these relationships apply. Here, we test if spatial and temporal variation in temperature affects genetic variation and plasticity of fitness and a key thermal tolerance trait (critical thermal maximum; CT_max_) at microgeographic scales using a metapopulation of *Daphnia magna* in freshwater rock pools. Specifically, we ask if (a) there is a microgeographic adaptation of CT_max_ and fitness to differences in temperature among the pools, (b) pools with greater temporal temperature variation have more genetic variation or plasticity in CT_max_ or fitness, and (c) increases in temperature affect the heritability of CT_max_ and fitness. Although we observed genetic variation and plasticity in CT_max_ and fitness, and differences in fitness among pools, we did not find support for the predicted relationships between temperature variation and genetic variation or plasticity. Furthermore, the genetic variation and plasticity we observed in CT_max_ are unlikely sufficient to reduce the impacts of climate change. CT_max_ plasticity was minimal and heritability was 72% lower when *D. magna* developed at the higher temperatures predicted under climate change. In contrast, the heritability of fitness increased by 53% under warmer temperatures, suggesting an increase in overall evolutionary potential unrelated to CT_max_ under climate change. More research is needed to understand the evolutionary and plastic potential under climate change and how that potential will be altered in future climates.

## Introduction

Species are already shifting their distributions, reducing their body size, and changing the timing of important life events in response to industrial-era climate change (Chen et al., 2011; Gardner et al., 2011; Socolar et al., 2017). Climate change is also causing many extirpations and has resulted in at least one extinction (Gynther et al., 2014; Román-Palacios & Wiens, 2020). As climate change accelerates, so too will its effects, including the potential extinction of up to 16% of species (Urban, 2015). Such large-scale extinction will substantially affect ecosystems and human well-being if we do not implement effective strategies to minimize biodiversity loss (Cardinale et al., 2012; Hooper et al., 2012). Consequently, biologists seek to predict which species and populations will be most vulnerable to climate change to help guide climate change conservation strategies.

Accurate vulnerability predictions are critical for implementing effective conservation under climate change. However, many studies evaluating climate change vulnerability could be inaccurate because they exclude important biological responses such as the potential for climatic tolerances to change via evolution or plasticity (Dawson et al., 2011; Urban et al., 2016). Evolutionary and plastic responses to recent climate change have been documented for many species, which suggests their importance in making accurate vulnerability predictions (De Meester et al., 2011; Gunderson & Stillman, 2015; Nadeau & Urban, 2019). Conversely, many populations are not responding via evolution or plasticity, even when those responses are expected (Evans et al., 2018; Vtipil & Sheth, 2020). Understanding which populations are likely to respond via plasticity or evolution could therefore improve vulnerability estimates and guide limited conservation resources to the most vulnerable populations.

A large body of research suggests that the degree of spatial and temporal climatic variation (e.g., daily, seasonal, and interannual) a population experiences can predict the population’s evolutionary or plastic potential (Botero et al., 2015; Diamond, 2017; Nadeau et al., 2017). Specifically, populations that experience more spatial (e.g., mountainous regions) or temporal climatic variation (e.g., temperate regions) might have higher evolutionary or plastic potential for three reasons. First, metapopulations that experience more climatic variation in space might have higher genetic variation in climatic-tolerance traits or fitness if each population experiences different climates and is adapted to the climate it experiences (i.e., the microgeographic adaptation hypothesis) (Huang et al., 2015; Richardson et al., 2014; Slatkin, 1987; Yeaman & Jarvis, 2006). Second, a population that experiences more temporal climatic variation among generations might also have higher genetic variation in climatic-tolerance traits or fitness if there is a fitness tradeoff between performance at high and low temperatures and therefore fluctuating selection maintains multiple genotypes (i.e., the fluctuating selection hypothesis) (Botero et al., 2015; Diamond, 2017; Huang et al., 2015; Tufto, 2015). In both cases, high genetic variation associated with high climatic variation could increase evolutionary potential by providing fuel for evolution under climate change (Bergland et al., 2014; Kelly et al., 2003; Mariac et al., 2016; Rodriguez-Trelles et al., 2013). Third, populations that experience more temporal climatic variation within generations might evolve more plastic climatic-tolerance traits if there is a tradeoff between fitness at high and low temperatures and a cost to plasticity (Botero et al., 2015; Tufto, 2015). Under these conditions, species evolve plasticity to match future environmental conditions when there is high environmental variation, but evolve to have less or no plasticity when there is less environmental variation. The predictability of environmental variation is also important because predictable variation provides a reliable cue for future conditions (Botero et al., 2015). Hence, populations that experience more climatic variation, especially if that variation is predictable, might be more plastic, and therefore fitness might be affected less by climate change (i.e., the adaptive plasticity hypotheses).

Despite the promise of predicting evolutionary and plastic potential with climatic variation, we still need to understand the scales at which these relationships apply. Most studies testing for relationships between climatic variation and climate change vulnerability compare species or populations across broad latitudinal gradients (e.g., temperate versus tropical) (Deutsch et al., 2008; Diamond, 2017; Hof et al., 2012; Krenek et al., 2012; Simon et al., 2015; Vasseur et al., 2014), where gene flow is likely low and therefore selection can act more effectively (Lenormand, 2002). Yet, climatic variation often differs at fine-spatial scales where gene flow can be higher. For example, temperature variation increases substantially from the soil to the canopy of tropical wet forests (Scheffers et al., 2017). Adaptation to such fine-scale climatic variation might occur despite gene flow, a phenomenon known as microgeographic adaptation (Richardson et al., 2014). If species exhibit microgeographic adaptation to climatic variation, we could make fine-scaled predictions of evolutionary and plastic potential, which could improve vulnerability estimates and the design of conservation strategies.

In addition, to predict evolutionary potential under climate change, we must understand if the heritability (i.e., the proportion of phenotypic variation that is due to genetics) of climatic-tolerance traits will change in the future. Heritability is often measured in a single laboratory environment or under current field conditions. However, heritability can change as environments change and populations evolve (Hoffmann & Merilä, 1999; Visscher et al., 2008). Hence, heritability measured under current conditions could misrepresent evolutionary potential in the future as climates change, and genotypes and species shift their distributions. In order to accurately predict species vulnerability to climate change, more research is clearly needed to understand how heritability might change in the future.

**Figure 1.**
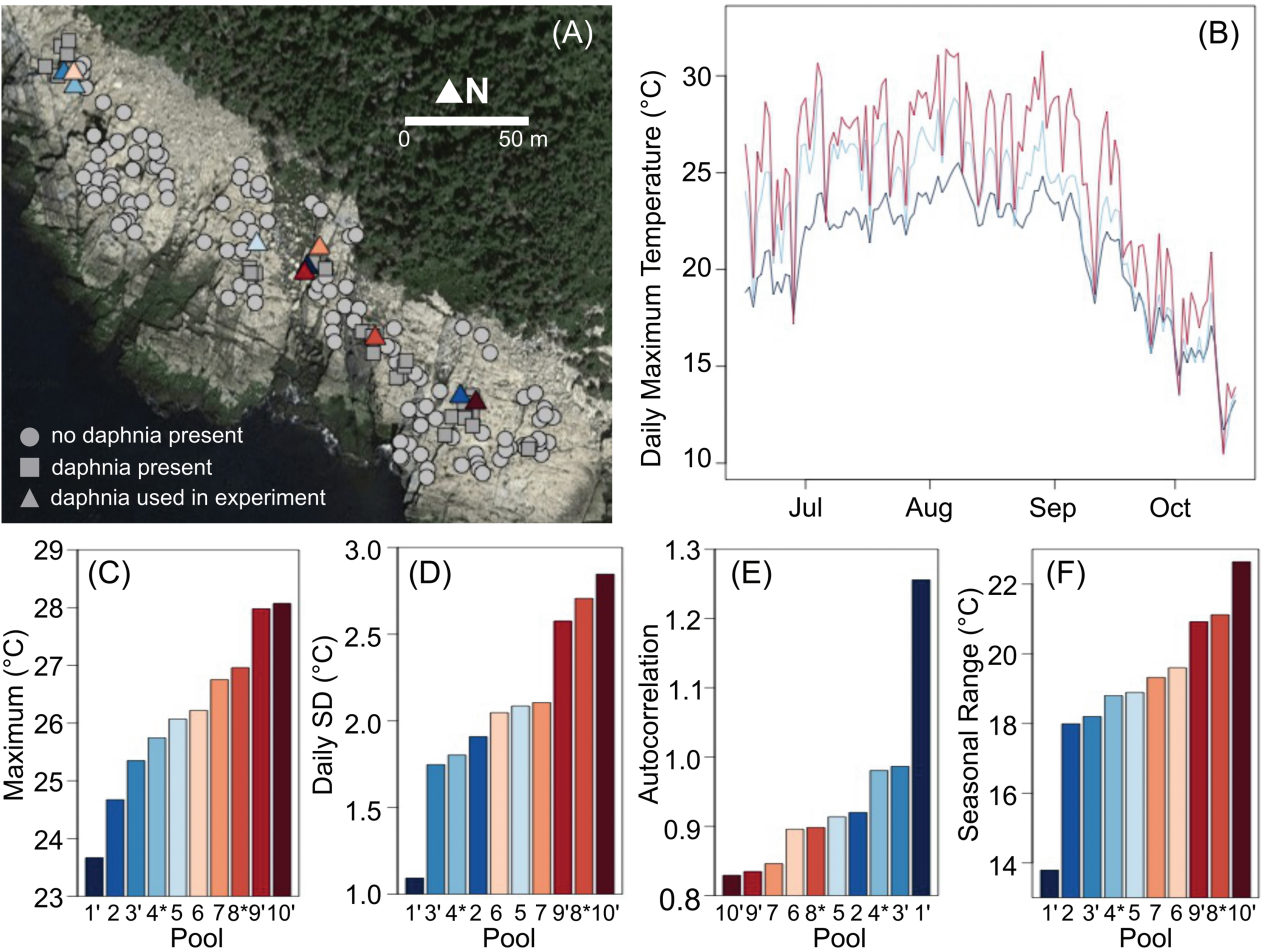
Differences in water temperature variation in 10 focal freshwater rock pools used to test for microgeographic adaptation in *Daphnia magna*. (A) A map of all freshwater rock pools in the study area. The color of the triangular symbols corresponds to the colors in (B–E). (B) Time series of daily maximum temperature between June 15 and October 15, 2018 from three pools (pool 1, dark blue; pool 4, light blue; and pool 9, red) with different amounts of temperature variation. Differences in (C) average daily maximum temperature during the hottest month (August), (D) daily variation in maximum temperature, (E) the predictability of maximum daily temperature (higher autocorrelation equals higher predictability), and (F) seasonal temperature range for each of the 10 focal pools. Pool labels followed by an apostrophe (‘) were only sampled for *D. magna* in 2018 and pool numbers followed by an asterisk (*) were only sampled in 2017, all other pools were sampled in both years.

Here, we test if fine-scale spatial and temporal variation in temperature affects genetic variation and plasticity of fitness (specifically, the intrinsic rate of natural increase) and a key thermal tolerance trait (critical thermal maximum; CT_max_) in a small (1.9 ha) rock-pool metapopulation of *Daphnia magna*. Maximum temperature differs substantially among pools separated by 1–250 m in our study area (Figure 1 and Supplementary Material S1). Moreover, temperature variation at different temporal scales (e.g., daily, seasonally) differs among pools, which provides an opportunity to test the fluctuating selection and adaptive plasticity hypotheses described above. We use lab measurements of fitness and CT_max_ to test (a) if there is microgeographic adaptation in CT_max_ and fitness to the temperature in each pool within the metapopulation, (b) if *D. magna* populations from pools with higher temporal temperature variation have higher broad-sense genetic variation in CT_max_ or fitness, (c) if *D. magna* from pools with higher temporal temperature variation or predictability have higher CT_max_ plasticity and less variation in fitness when raised under different temperatures, and (d) how the genetic and nongenetic components of CT_max_ and fitness heritability are affected by increased temperature.

## Methods

### Study system

*Daphnia magna* is a small freshwater crustacean with a Holarctic distribution. *Daphnia magna* reproduces clonally approximately once per week (depending on temperature) and sexually reproduces periodically. We focus on a metapopulation of *D. magna* in freshwater rock pools on Schoodic Point in Acadia National Park, Maine, USA. The freshwater rock pools on Schoodic Point are depressions in the bedrock that fill with rainwater. The pools generally remain inundated all year and freeze in the winter (Nadeau et al., 2022). *Daphnia magna* persists in pools throughout the winter as sexually produced resting eggs. *Daphnia magna* likely disperses among nearby pools when the pools overflow during heavy rain events (Vanschoenwinkel et al., 2008) and disperses longer distances if transported by gulls (family: Laridae), which regularly bathe in the larger pools (Simonis & Ellis, 2014). However, we do not have data on the amount of dispersal, gene flow, or genetic structure of *D. magna* in the focal metapopulation (see Discussion for more details).

Temperature variation differs significantly among pools due to differences in water depth and solar exposure (Figure 1; Nadeau et al., 2022). We predicted that *D. magna* would be adapted to differences in temperature variation among the pools for several reasons. First, *D. magna* is under strong selection from high temperatures. Indeed, *D. magna* is often adapted to maximum temperatures experienced at regional scales (Fields et al., 2015; Seefeldt & Ebert, 2019; Yampolsky et al., 2014) and has evolved rapidly in response to temperature changes caused by climate change and urbanization (Brans et al., 2017; De Meester et al., 2011; Geerts et al., 2015). Second, *D. magna* populations are often genetically structured at microgeographic scales (De Meester, 1996; Haag et al., 2005, 2006) and other *Daphnia* species show evidence of local adaptation at very fine spatial scales (Declerck et al., 2001). Third, the focal rock pools are shallow (mean depth = 24 cm) and well mixed by coastal winds. Therefore, water temperature is often homogeneous throughout a pool, which makes local adaptation more likely because species cannot avoid extreme temperatures with behavioral thermoregulation (Gunderson & Stillman, 2015). Hence, *D. magna* is a good candidate to observe microgeographic adaptation.

### Temperature variation in focal pools

We focused on 10 pools identified a priori that differed in temperature variation (Figure 1). We measured the daily maximum and mean temperature in each pool between June 15 and October 15, 2018 (the primary growing season for *D. magna* in our study site) using temperature data loggers (models: HOBO Pendant UA-001-08 or Onset Hobo U20L) placed in the deepest part of the pool and covered with a rock to block direct sunlight. We used these data to calculate the average daily mean temperature for the entire period, the monthly average of daily maximum temperature in the hottest month (August), the monthly standard deviation of daily maximum temperature across the season (i.e., within-generation variation), the predictability of daily maximum temperature (i.e., within-generation predictability), and the seasonal temperature range (i.e., among-generation variation; Figure 1). We mostly focus on maximum temperature because pools differ the most in maximum temperature, we expect *D. magna* CT_max_ to be associated with maximum temperature, and we know CT_max_ is plastic in *D. magna* (Yampolsky et al., 2014). Although data from all study pools are only available in 2018, data from a subset of pools in other years suggest that the rank order of pools based on each temperature variable is similar among years (Supplementary Material S1). Moreover, the primary factors causing temperature differences among pools—solar exposure and water depth—are also consistent among years (Nadeau et al., 2022). Hence, the temperature differences among pools, and therefore the selective pressures of interest, are likely consistent among years.

We used generalized least squares to estimate the daily standard deviation and predictability of maximum temperature for each pool. We fit models independently to the temperature time series for each pool using the “*gls*” function in the “*nlme*” package (Pinheiro et al., 2023) in R version 3.6.0. We fit a quadratic model with daily maximum temperature as the response variable, Julian date as a quadratic covariate, and specified the temporal correlation structure using a Gaussian variogram model. The quadratic model removes the seasonal component of the temperature variation before estimating the daily standard deviation and predictability. We estimated the daily standard deviation from model residuals and predictability as the range of the variogram model (i.e., the number of days over which temperature measurements are autocorrelated). The focal pools differed substantially in all measures of temperature variation (Figure 1). However, the measures of temperature variation were highly correlated (Figure 1). Hence, if we observe microgeographic adaptation, it would not be possible to discern which measure of temperature was driving selection. Nonetheless, our multiple hypotheses are differentiated by the response variables (see below) and not the temperature variables. Hence, our study could still differentiate among the hypotheses, even if the exact mechanism of adaptation is unknown.

### *Daphnia* collection and clonal maintenance

We collected *D. magna* from the 10 focal pools using either a dip net or a plastic pipette. In 2017, we collected one to eight adult females from each of the six pools. In 2018, we collected 25 adult females from each of eight pools, including four pools sampled in 2017. We assume that each female is a separate clone. This assumption may not be strictly true due to clonal reproduction. However, we collected females shortly after ephippia hatched in the spring, which is when clonal variation is highest. We kept each female in the lab in separate 100-ml specimen cups filled with 80 ml of water from a local freshwater rock pool that we filtered through 500-µm mesh to remove invertebrates. We kept each cup at room temperature under natural light and added algae daily. We haphazardly selected two neonates following brooding to continue the clonal line in the lab.

The two neonates from each clone grew in separate 120-ml specimen cups filled with 100 ml of filtered and sterilized water from a local stream. We kept each cup in a 20 °C incubator with a 16:8 light–dark cycle. Every 2–3 days, we fed each individual 200 µl of algae culture with a standardized density of 37.5 × 10^6^ cells/ml and checked for newborns. If we observed newborns, we haphazardly separated two neonates to continue the clonal line. We repeated this process for at least two generations to reduce maternal effects.

### CT _max_ and fitness assays

After growing the clones in the lab for at least two generations, we split a brood from each clonal line and put two to three neonates in a 20 °C incubator and two to three neonates in a 25 °C incubator in separate 120-ml specimen cups. We chose 20 °C because the average daily mean temperature between June and October in the 10 focal pools was 20.0 °C (*SD* = 3.5 °C). We chose 25° to represent 5 °C of potential warming, which is the predicted change in air temperature under a high emissions scenario in our study area by 2100 (Lynch et al., 2015). We grew all clones at both temperatures until they were 14–30 days old, feeding them as described above.

We recorded the age of the female and the size of the brood for the first and second reproductive events. We estimated fitness as the intrinsic rate of natural increase (*r*) for each individual using the Lotka–Euler equation (Van Doorslaer et al., 2007, 2010). We only included individuals that reproduced at least once (i.e., mature females) and that we monitored for at least 15 days when analyzing fitness to provide ample opportunity for the female to mature and produce two broods. In total, we measured fitness on 580 individuals that originated from 129 clones, with an average of 12.9 (*SD* = 5.6; Supplementary Table S2) clones per pool. Simulations suggest that the number of individuals we used per clone did not alter our results (Supplementary Material S5).

We measured CT_max_ on the mature females from the above fitness experiments by putting each female in a 5 ml glass beaker filled with water and suspended in a water bath with an initial temperature of 22.5 °C. We raised the temperature to 0.5 °C/min and recorded the temperature when each individual lost equilibrium and sank to the bottom. We measured the CT_max_ of an average of 15.2 (*SD* = 5.5) individuals per trial including a haphazard assortment of individuals from each developmental temperature. In total, we measured CT_max_ on 563 mature females that originated from 131 clones, with an average of 13.1 (*SD* = 5.6; Supplementary Table S2) clones per pool. We measured CT_max_ on fewer females than we measured fitness because some females died before we conducted CT_max_ assays (see Supplementary Material S2 for final sample sizes).

### Testing hypotheses and estimating heritability

We compared three sets of Bayesian linear mixed-effects models to evaluate the hypotheses described above and evaluate temperature effects on heritability. In all models, we pooled the data from 2017 and 2018, but included a fixed effect for each year to account for potential differences among years. We compared models using approximate leave-one-out cross-validation in the “*loo*” package in R (Vehtari et al., 2017). We used the “*loo*” function to calculate expected log predictive densities (ELPDs), which are used to compare models, and the “*loo_compare*” function to compare models based on the ELPD values for each model. We assumed a model received some support if the difference in ELPD relative to the top model was at least twice the SE of the difference in the ELPD between the models. We also evaluated the 95% credible intervals for the relevant coefficients to assess support for each hypothesis (see below). We fit all models in JAGs using the “*jags*” function in the “*R2jags*” package in R version 3.6.0. We used vague normal priors (mean = 0; precision = 0.001) for all coefficients. We used vague uniform priors (range: 0–100) for variance parameters. We used the following MCMC settings depending on model complexity: (M1-0 to M1-3) 10,000 iterations, a burn-in of 5,000 iterations, and retained every 5th draw; (M1-4) 30,000 iterations, a burn-in period of 25,000 iterations, and retaining every 5th draw; and (M2-0 to M2-3 and M3-0 to M3-3) 75,000 iterations, a burn-in period of 25,000 iterations, and retained every 50th draw. These MCMC settings resulted in well-mixed chains as assessed visually and by Gelman–Rubin statistics < 1.1. Bayesian *p* values from posterior predictive checks of the means and variances (i.e., goodness of fit tests) of the best-fitting models (Table 1) were between 0.493 and 0.603, which suggests the models fit the data well (Hobbs & Hooten, 2015).

**Table 1.** Model comparisons to evaluate three hypotheses relating temperature variation to evolutionary and plastic potential and to evaluate the effects of temperature on the heritability of critical thermal maximum (CT_max_) and fitness in a *Daphnia magna* metapopulation. We used approximate leave-one-out cross-validation for model comparison. *P*_eff_ is the effective number of parameters, ELPD is the expected log predictive density, ∆ELPD is the difference in ELPD from the best-fitting model where larger values indicate higher model support, and ∆ELPD SE is the standard error in ∆ELPD. We assumed a model received some support if ∆ELPD was twice as large as ∆ELPD SE. See the main text and Supplementary Data S3 for model descriptions.

Model and hypothesis	Fitness				CTmax			
	*P*_eff_	ELPD	∆ELPD	∆ELPD SE	*P*_eff_	ELPD	∆ELPD	∆ELPD SE
Microgeographic and fluctuating selectin hypotheses								
M1-0: no genetic variation	3.57	579.2	−34.8	10.69	27.8	−204.5	−8.1	4.41
M1-1: genetic variation	76.3	610.8	−3.2	3.14	**66.6**	−**196.4**	**0.0**	**0.00**
M1-2: CTmax or fitness differs among pools	72.9	612.9	−1.1	1.11	67.4	−198.0	−1.6	0.71
M1-3: microgeographic adaptation	**72.2**	**614.0**	**0.0**	**0.00**	69.0	−197.5	−1.1	0.92
M1-4: genetic variation differs among pools (fluctuating selection)	84.3	608.5	−5.5	4.50	83.4	−200.6	−4.1	3.69
Adaptive plasticity hypotheses								
M2-0: no plasticity	87.7	1,073.3	−62.4	11.80	59.7	−395.2	−32.1	8.64
M2-1: plasticity	**99.8**	**1,135.7**	**0.0**	**0.00**	**63.8**	−**363.1**	**0.0**	**0.00**
M2-2: adaptive plasticity associated with temperature variation	101.4	1,133.9	−1.8	0.64	64.6	−363.5	−0.4	1.46
M2-3: adaptive plasticity associated with temperature predictability	100.5	1,134.5	−1.2	0.91	64.9	−363.5	−0.4	1.27
Temperature effects on heritability components								
M3-0: temperature does not affect genetic or nongenetic variance	**103.3**	**1,148.6**	**0.0**	**0.00**	66.5	−362.8	−3.2	3.50
M3-1: temperature affects non-genetic variance	104.6	1,148.3	−0.3	3.82	66.7	−364.4	−4.8	3.34
M3-2: temperature affects genetic variance	168.5	1,146.6	−2.0	11.20	**86.7**	−**359.6**	**0.0**	**0.00**
M3-3: temperature affects genetic and non-genetic variance	169.9	1,144.7	−3.8	11.23	88.8	−360.5	−0.9	0.96

The top models are shown with bold text for each response variable.

#### The microgeographic adaptation hypothesis

**Figure 2.**
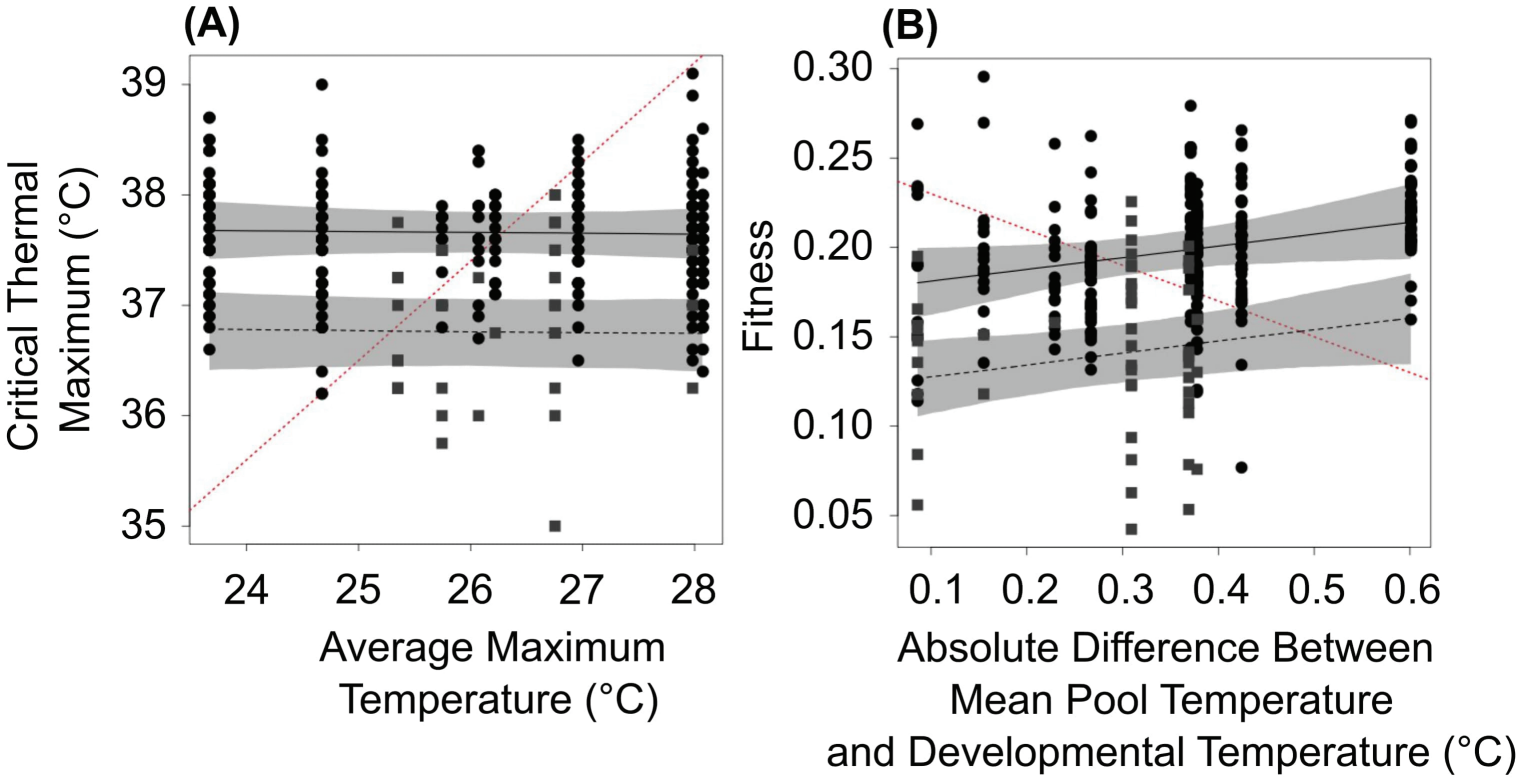
A test of predictions from the microgeographic adaptation hypothesis for (A) critical thermal maximum and (B) fitness of *Daphnia magna* from 10 freshwater rock pools. Black circles show data from 2018 and gray squares show data from 2017. Points show measurements of critical thermal maximum (A) or fitness (B) from *D. magna* that developed at 20 °C in the laboratory. The black lines (solid 2018, dashed 2017) show the median, and the gray shaded area shows the 95% credible interval of the estimated relationship from model M1-3 (see main text). The red dashed line shows the direction of the predicted relationship.

This hypothesis predicts that *D. magna* from pools with higher maximum water temperatures will have higher CT_max_ (Figure 2A) and that *D. magna* from pools with a mean temperature most similar to the developmental temperature in the laboratory will have the highest fitness. To test the CT_max_ prediction, we analyzed the CT_max_ data from individuals that developed at 20 °C (i.e., current average temperature). We excluded individuals that developed at 25 °C to simplify the modeling. We first fit a null model with CT_max_ as the response variable, year as a fixed effect, and a random effect for the C_Tmax_ trial, but without effects of clone or pool (model M1-0, Supplementary Material S3). This null model assumes no genetic variation in CT_max_ among clones. We fit the year as a fixed effect because there were too few levels (*n* = 2) to estimate the random-effect variance. We next added a clone random effect to the null model, assuming genetic variation in CT_max_, but no variation among pools or microgeographic adaptation (model M1-1, Supplementary Material S3). We then evaluated if CT_max_ differed among pools by adding a pool random effect to model M1-1 (model M1-2, Supplementary Material S3). Last, we added the average maximum pool temperature in the warmest month as a fixed effect to model M1-2 to evaluate if CT_max_ increased with maximum pool temperature as predicted (model M1-3, Supplementary Material S3). To test the fitness prediction, we fit models M1-0 through M1-3 with fitness as the response variable and excluding the trial random effect, which is not relevant to measures of fitness. In model M1-3, we used the absolute difference between the mean pool temperature and 20 °C as the temperature-fixed effect.

#### The fluctuating selection hypothesis

**Figure 3.**
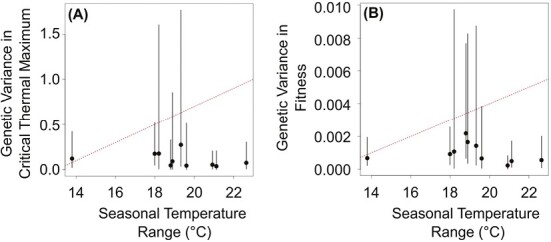
Tests of the fluctuating selection hypothesis for (A) critical thermal maximum and (B) fitness of *Daphnia magna* from 10 freshwater rock pools. Red dotted lines show the direction of the predicted relationship. Black points and error bars represent the median and 95% credible interval of genetic variance estimated from model M1-4 (see main text).

This hypothesis predicts that pools with more among-generation temperature variation (i.e., seasonal temperature variation) will have higher genetic variation in CT_max_ and fitness (Figure 3). We do not know if *D. magna* exhibits the tradeoff required for this relationship (see Introduction). However, local adaptation in space requires a similar tradeoff and previous studies have demonstrated local adaptation in *D. magna* upper temperature tolerances (Fields et al., 2015; Seefeldt & Ebert, 2019; Yampolsky et al., 2014). To test this prediction, we modified model M1-1 such that the variance of the clone random effect (i.e., the genetic variance) differed among pools (model M1-4, Supplementary Material S3). We did not explicitly test whether genetic variance was related to temperature variation in each pool because models associating genetic variance (i.e., the variance of the clone random effect) with seasonal temperature variation failed to converge. However, we plotted estimates of genetic variation from model M1-4 versus the seasonal temperature range in each pool to qualitatively evaluate if genetic variation was higher in pools with higher seasonal temperature variation (Figure 3).

#### The adaptive plasticity hypotheses

**Figure 4.**
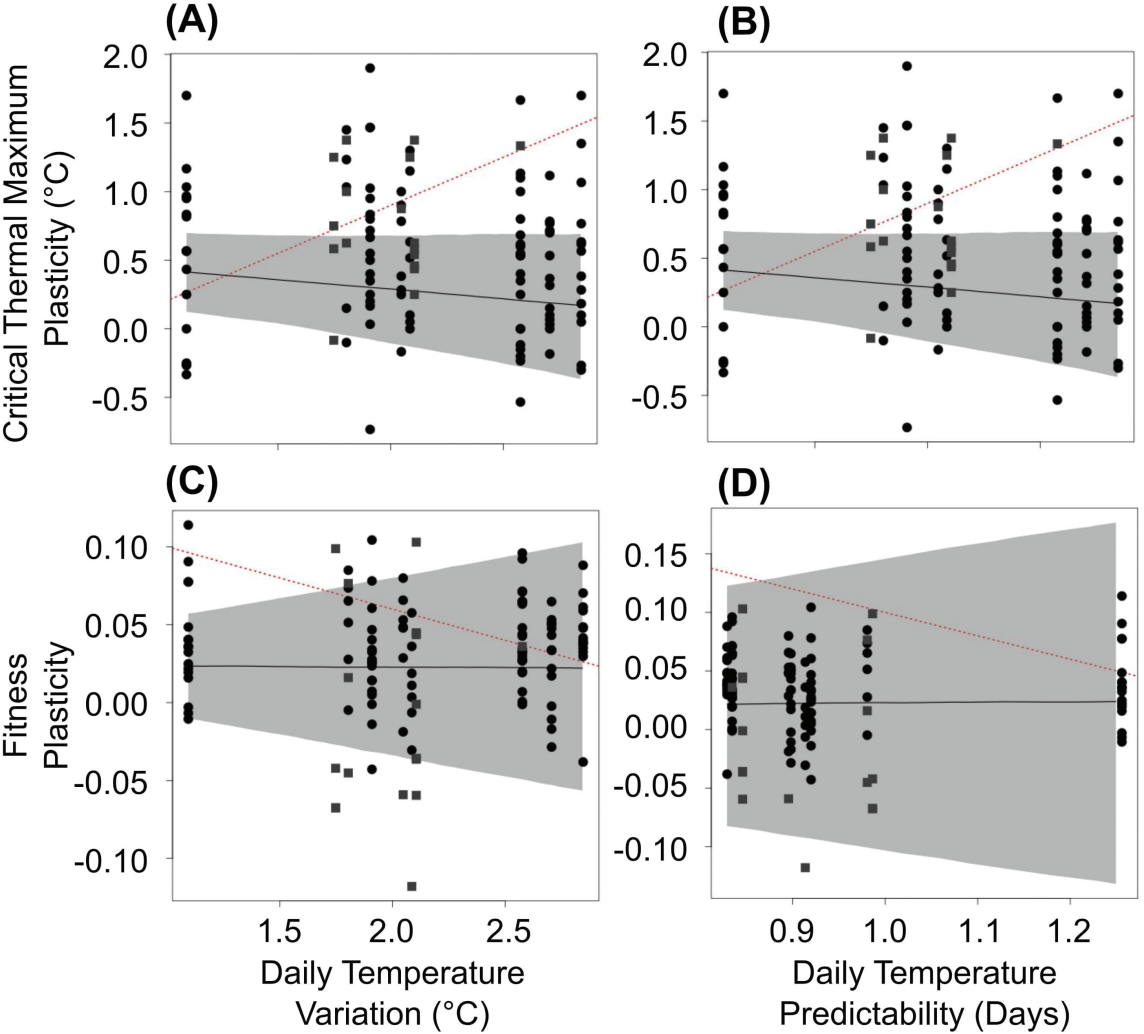
Tests of the adaptive plasticity hypothesis for (A and B) critical thermal maximum and (C and D) fitness of *Daphnia magna* from 10 freshwater rock pools. Red-dotted lines show the direction of the predicted relationship. Black circles (2017) and gray squares (2018) show the difference in the average critical thermal maximum or fitness for each clone when they developed at 20 °C and 25 °C (i.e., plasticity). The black line and gray shaded areas show the median and 95% credible interval of the predicted relationship from models (A and C) M2-2 and (B and D) M2-3.

These hypotheses predict CT_max_ plasticity will be higher and fitness plasticity will be lower (i.e., plasticity will allow *Daphnia* to have high fitness at both temperatures) in pools with more within-generation (i.e., daily) temperature variation, especially if that variation is predictable (Figure 4). We analyzed CT_max_ and fitness data from individuals that developed at 20 °C and 25 °C together to test for plasticity, and if variation in plasticity among populations correlated with temperature variation among pools of origin. We first fit a null model with a year fixed effect and clone, pool, and trial random effects (CT_max_ only) to represent the scenario where there is no plasticity in C_Tmax_ or fitness (model M2-0, Supplementary Material S3). We then added a fixed effect for lab developmental temperature (20 °C or 25 °C) to the null model to evaluate if there is plasticity (model M2-1, Supplementary Material S3). We used two different models to evaluate if plasticity was associated with temperature variation. First, we added a daily temperature variation fixed effect and an interaction between daily temperature variation and developmental temperature to model M2-1 to evaluate if plasticity was associated with daily temperature variation as predicted (model M2-2, Figure 2A and B, Supplementary Material S3). Last, we added a daily temperature predictability fixed effect and an interaction between daily temperature predictability and developmental temperature to model M2-1 to evaluate if plasticity was associated with daily temperature predictability as predicted (model M2-3, Figure 2B and D).

#### Effects of temperature on heritability

To evaluate how increased temperatures affected the genetic and nongenetic components of phenotypic variation, we analyzed CT_max_ and fitness data from individuals that developed at 20 °C and 25 °C using modified versions of model M1-1. Note that model M1-1 does not include any population-level effects. Hence, for the sake of estimating heritability we are treating the entire metapopulation as a whole. We first modified model M1-1 such that the intercept and year fixed effect could differ depending on environmental temperature, while keeping the variance of the clone random effect (i.e., the genetic variance) and error variance (i.e., the nongenetic variance) the same for both developmental temperatures (model M3-0, Supplementary Material S3). This model represents the null model of no effect of developmental temperature on either genetic or nongenetic components. Second, we modified model M3-0 such that the nongenetic variance could vary with developmental temperature (model M3-1, Supplementary Material S3). Third, we modified model M3-0 such that the genetic variance could vary with developmental temperature (model M3-2, Supplementary Material S3). Last, we modified model M3-0 such that both the genetic and nongenetic variance could vary with developmental temperature (model M3-3, Supplementary Material S3). We estimated heritability from model M3-3 as the ratio of genetic variance (i.e., the variance of the clone random effect) and total phenotypic variance (i.e., the variance of the clone random effect plus the error variance).

## Results

### Microgeographic adaptation hypothesis

When *D. magna* developed at 20 °C, CT_max_ ranged between 35.0 °C and 39.1 °C among the 296 individuals tested (mean = 37.5 °C, *SD* = 0.6 °C). CT_max_ was 0.9 °C (95% credible interval [CI] = 0.6 °C to 1.2 °C) higher in 2018 relative to 2017. The best-fitting model for CT_max_ (M1-1) included genetic variation in CT_max_ among clones, but no variation in CT_max_ among pools or microgeographic adaptation (Table 1; Figure 2A). The models suggesting no genetic variation (M1-0) and microgeographic adaptation (M1-3) also received some support (Table 1). However, the relationship between CT_max_ and maximum temperature in the pool of origin was slightly negative (i.e., opposite of the predicted relationship) and the 95% CI of the relationship overlapped zero (95% CI = −0.080 to 0.067; Figure 2A). Simulations suggest this result was unlikely due to a lack of power to detect a biologically meaningful relationship between CT_max_ and the maximum temperature in each pool (Supplementary Material S4). Hence, results suggest potential genetic variation in CT_max_ among clones in the metapopulation that is not associated with differences in maximum temperature among pools as predicted by the microgeographic adaptation hypothesis (Figure 2A).

When *D. magna* developed at 20 °C, fitness ranged between 0.042 and 0.296 among the 303 individuals tested (mean = 0.189, *SD* = 0.041). Fitness was 0.056 (95% CI = 0.037 to 0.074) higher in 2018 relative to 2017. The best-fitting model for fitness (M1-3) suggested microgeographic adaptation to temperature (Table 1; Figure 2B). However, the relationship between pool temperature and fitness was the opposite of the predicted relationship (Figure 2B). Models suggesting no differences in fitness among pools (M1-1) also received support, but models suggesting no genetic variation in fitness were not strongly supported (Table 1). Hence, results suggest genetic variation in fitness among clones in the metapopulation, but little support for the microgeographic adaptation hypothesis (Figure 2B), although there could be adaptation related to factors other than mean temperature.

### Fluctuating selection hypothesis

The model including differences in genetic variation of CT_max_ or fitness among pools (M1-4) received some support (Table 1). However, genetic variation did not increase with the seasonal temperature range in the pool of origin as predicted (Figure 3). Hence, genetic variation is not higher in pools with more seasonal temperature variation as predicted by the fluctuating selection hypothesis (Figure 3).

### Adaptive plasticity hypotheses

When we raised *D. magna* at 25 °C, CT_max_ ranged between 36.2 °C and 39.5 °C among the 266 individuals tested (mean = 38.1 °C, *SD* = 0.5 °C). The best-fitting model for CT_max_ (M2-1) included an effect of developmental temperature (i.e., plasticity), but no relationship between plasticity and the amount or predictability of temperature variation in the pool of origin (Table 1). CT_max_ was 0.4 °C (95% CI = 0.3 to 0.5) higher when *D. magna* developed at 25 °C relative to 20 °C. The models including an interaction between developmental temperature and temperature variation (M2-2) or predictability (M2-3) in the pool of origin were also well supported (Table 1). However, the 95% credible intervals for the parameters describing the interaction with temperature variation (95% CI = −0.222 to 0.051) and predictability (95% CI = −0.252 to 0.848) overlapped zero (Figure 4A and B). Simulations suggest this result was unlikely due to a lack of power to detect a biologically meaningful relationship between CT_max_ plasticity and the temperature variation in each pool (Supplementary Material S4). Hence, our results indicate plasticity in CT_max_, but no effect of temperature variation or predictability on plasticity as predicted by the adaptive plasticity hypotheses (Figure 4A and B).

When we raised *D. magna* at 25 °C, fitness ranged between 0.000 and 0.376 among the 266 individuals tested (mean = 0.224, *SD* = 0.054). The best-fitting model for fitness (M2-1) included an effect of developmental temperature (i.e., plasticity), but no relationship between plasticity and the amount or predictability of temperature variation in the pool of origin (Table 1). Fitness was 0.029 (95% CI = 0.024 to 0.035) higher when *D. magna* developed at 25 °C relative to 20 °C. The model including an interaction between developmental temperature and temperature predictability (M2-3) in the pool of origin also had some support (Table 1). However, the 95% credible interval for the parameters describing the interaction with temperature predictability (95% CI = −0.022 to 0.051) overlapped zero (Figure 4D). Simulations suggest this result was unlikely due to a lack of power to detect a biologically meaningful relationship between fitness plasticity and the temperature variation in each pool (Supplementary Material S4). Hence, our results indicate plasticity in fitness, but no effect of temperature variation or predictability on the evolution of site-specific plasticity as predicted by the adaptive plasticity hypotheses (Figure 4C and D).

### Temperature effects on heritability

**Figure 5.**
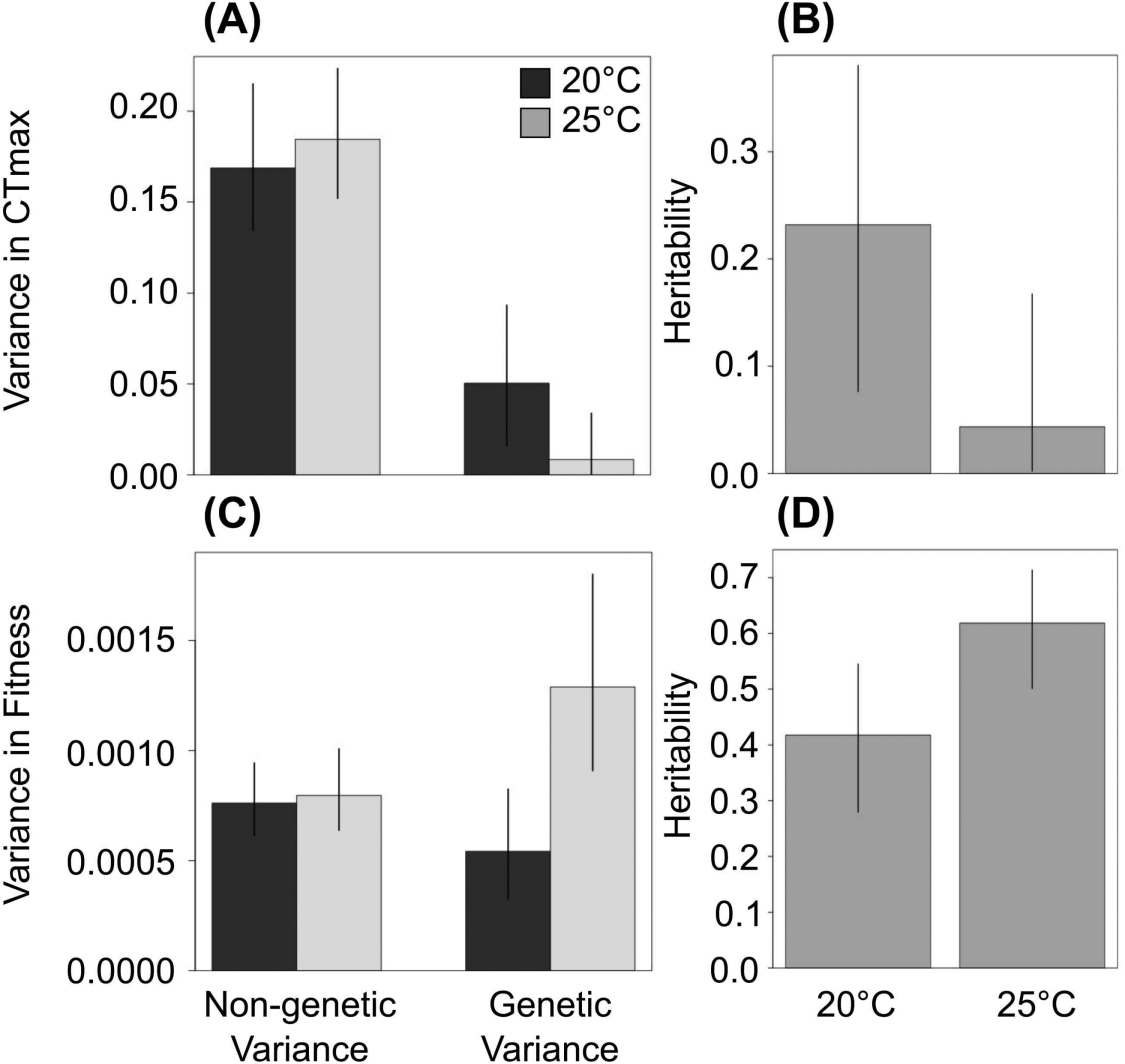
Effects of developmental temperature on estimates of nongenetic and genetic variance of (A) critical thermal maximum and (C) fitness and heritability of (B) critical thermal maximum and (D) fitness from model M3-3. The bars and error bars show the medians and 95% credible intervals, respectively.

All four models evaluating how the components of phenotypic variance were affected by developmental temperature received some support for both CT_max_ and fitness (Table 1). Based on model M3-3 for CT_max_, temperature had little effect on the nongenetic component of CT_max_ variance (95% CI of the difference in nongenetic variance between temperatures = −0.068 to 0.038), but caused a 75% decrease in the genetic variance (95% CI of the difference in genetic variance between temperatures = 0.002 to 0.085), which resulted in a 72% decrease in the heritability of CT_max_ (95% CI of the difference in heritability between temperatures = 0.006 to 0.343; Figure 5). Based on model M3-3 for fitness, temperature had little effect on the nongenetic component of fitness variance (95% CI of the difference in nongenetic variance between temperatures = −0.0002 to 0.0003), but caused a 150% increase in the genetic variance (95% CI of the difference in genetic variance between temperatures = 0.0003 to 0.0013), which resulted in a 53% increase in the heritability of fitness (95% CI of the difference among temperatures = 0.034 to 0.375; Figure 5). Hence, the results suggest that increases in developmental temperature reduced the heritability of CT_max_ by reducing genetic variance, but increased the heritability of fitness by increasing genetic variance (Figure 5).

## Discussion

We evaluated whether microgeographic variation in temperature predicted the evolutionary and plastic potential of thermal tolerances and fitness. Temperature variation differed substantially among the ten focal freshwater rock pools. For example, some pools located less than 1 m apart differed in absolute maximum temperature in 2018 by 7.1 °C, which is similar to the temperature difference expected over a 1,200 m change in elevation or a 131-km change in latitude in our study region. Although we saw some evidence of microgeographic adaptation in fitness, the differences in temperature variation among pools were not associated with the evolutionary or plastic potential of thermal tolerance or fitness as predicted by three hypotheses that often apply at larger spatial scales (Botero et al., 2015; Diamond, 2017; Nadeau et al., 2017).

Despite finding little support for the tested hypotheses, we did observe developmental plasticity and genetic variation in thermal tolerances and fitness, which suggests some ability for *D. magna* to respond to increased temperatures. However, our results also suggest those responses might be limited. We observed a 0.4 °C increase in CT_max_ with a 5 °C increase in developmental temperature, which is similar to that observed in a global review of CT_max_ plasticity, but low for crustaceans included in that review (Gunderson & Stillman, 2015). Such small effects are unlikely to reduce the vulnerability of most species to climate change (Gunderson & Stillman, 2015; Gunderson et al., 2017). Plasticity might be higher in nature where fluctuating temperatures can result in acclimation effects. However, studies in *Drosophila* suggest these effects are small and unlikely to decrease climate change vulnerability (van Heerwaarden et al., 2016b).

We also observed evidence for genetic variation that could facilitate thermal tolerance evolution under climate change. When *D. magna* developed at 20 °C, the heritability of CT_max_ was similar to the average (0.28, 95% CI = 0.19 to 0.39) from a global review of heritability in upper thermal tolerances (Diamond, 2017). However, when *D. magna* developed at 25 °C, heritability decreased by 72% due to decreases in genetic variance. Consequently, the evolutionary potential for CT_max_ might decrease substantially as temperatures warm. In contrast, heritability for fitness—the best overall predictor of evolutionary potential (Walsh, 2022)—was high and increased significantly under warmer temperatures, suggesting hidden genetic variation for traits other than CT_max_ and an increased capacity for adaptation in general.

Changes in heritability among environments, including among different temperatures, are common. However, the direction of heritability changes in response to environmental changes is seldom predictable (Hoffmann & Merilä, 1999). For example, studies have observed both increases (Sisodia & Singh, 2009; van Heerwaarden et al., 2016a), decreases (Bennington & McGraw, 1996; Chirgwin et al., 2015; Ketola et al., 2012; Morgan et al., 2020), and no change (Bairos-Novak et al., 2021) in genetic variation, phenotypic variance, or heritability under increased temperatures. More research is needed to understand the effects of climate change on heritability to facilitate predictions of when and where we might expect evolution to alter species’ responses. Moreover, these results highlight the need to estimate heritability in projected future environments to accurately estimate evolutionary potential (Chirgwin et al., 2015; van Heerwaarden et al., 2016b).

The lack of predictable microgeographic adaptation in *D. magna* in our study system is somewhat surprising given what we know about the evolutionary potential of *D. magna* and population genetic structure in other parts of *D. magna’s* range. *Daphnia magna* can evolve rapidly in response to differences in temperature. Experiments with *D. magna* in lab and field mesocosms that increased temperatures by 4 °C demonstrated rapid evolution of population growth rate and size at maturity in as little as three months (De Meester et al., 2011; Van Doorslaer et al., 2009a, 2010). *Daphnia magna* is also one of the only species with documented CT_max_ evolution in response to recent climate change in nature (Geerts et al., 2015), and CT_max_ has also evolved in response to urban heat island effects (Brans et al., 2017). Moreover, *D. magna*’s CT_max_ is locally adapted to maximum temperatures across its range in Afro-Eurasia (Seefeldt & Ebert, 2019; Yampolsky et al., 2014). Genetic studies also commonly reveal population differentiation at fine-spatial scales (Haag et al., 2005, 2006; Orsini et al., 2012, 2013; Vanoverbeke & De Meester, 1997). For example, *D. magna* populations that occur in a very similar freshwater rock pool ecosystem show high levels of genetic differentiation even among pools just a few meters apart (Haag et al., 2005, 2006). This population differentiation is often associated with low gene flow and selective differences in local environments (Haag et al., 2006; Orsini et al., 2013). Last, even if dispersal among populations is high, gene flow that could erode microgeographic adaptation might be limited by the predominance of locally adapted genotypes that monopolize resources and limit the effective immigration of foreign genotypes (Boileau et al., 1992; De Meester et al., 2002, 2016). Indeed, multiple studies with *D. magna* have demonstrated this phenomenon (De Meester et al., 2002; Orsini et al., 2013; Van Doorslaer et al., 2009b). Together, these aspects of *D. magna* populations suggest microgeographic adaptation to temperature and temperature variation should be likely.

Why then did we not observe relationships between temperature variation and temperature tolerances as predicted? One reason might be the well-known effect of founder events and genetic drift in structuring *Daphnia* metapopulations (Haag et al., 2005, 2006; Orsini et al., 2013). In other freshwater rock pool metapopulations, approximately 16% of *D. magna* populations go locally extinct annually (Pajunen & Pajunen, 2003). Recolonization often occurs by just one to three individuals that rapidly increase in abundance through clonal reproduction, which results in strong founder effects (Ebert et al., 2002; Haag et al., 2005). Although these founder effects can result in population differentiation (Haag et al., 2005, 2006), they do not result in microgeographic adaptation because the founders are randomly selected from the metapopulation and populations go extinct prior to gaining the necessary genetic variation for adaptation (Haag et al., 2006). Such metapopulation dynamics might therefore limit microgeographic adaptation in our study system. However, local extirpation rates are likely much lower in our system, which limits the impact of repeated founder effects (Haag et al., 2005). Between 2016 and 2019, we did not observe any extirpation of *D. magna* populations in our study area, although extirpation and re-colonization could occur between our observations, and therefore extirpation events could have gone unobserved.

Gene flow reinforced by hybrid vigor could also prevent microgeographic adaptation to temperature variation in our study system. Dispersal, especially among nearby pools (including some focal pools in this study), is likely high due to the overflow of water from one pool into adjacent pools during heavy rain events (Vanschoenwinkel et al., 2008). Dispersal among more distant pools might also be high if the gulls that we regularly observed bathing in the pools transport *D. magna* adults and ephippia among pools. Indeed, gulls are a significant dispersal agent for many other rock pool invertebrates on nearby Appledore Island, Maine (Simonis & Ellis, 2014). Our attempts to measure this longer-distance dispersal using 20 uninhabited artificial rock pools in 2016 failed to document *D. magna* dispersal, despite documenting the dispersal of other *Daphnids*. It is unclear whether this suggests longer-distance *D. magna* dispersal is infrequent, or if our methods were inadequate. However, even if dispersal among more distant pools is infrequent, effective gene flow might be high due to hybrid vigor. Infrequent dispersal can result in high effective gene flow in inbred populations if hybrids of inbred residents and immigrants have a strong fitness advantage (Ebert et al., 2002; Haag et al., 2005; Ingvarsson & Whitlock, 2000). In other *D. magna* metapopulations, hybrid vigor was estimated to increase the effective rate of gene flow approximately 35 times above what would be predicted by the number of immigrants alone (Ebert et al., 2002). Genomic analysis of *D. magna* individuals from our study system suggests strong inbreeding (D. Ebert, personal communication). Hence, hybrid vigor might significantly enhance gene flow in our study system.

Last, weak or variable selection might also result in a lack of microgeographic adaptation. Water temperatures never exceeded *D. magna* CT_max_ in any of our focal pools and are often more than 5 °C below CT_max_. Therefore, selection might not be strong for higher CT_max_ in warmer pools. However, if CT_max_ represents a general measure of warm tolerance that also applies at lower temperatures, the temperature may not need to exceed CT_max_ to impose strong selection. Moreover, temperatures vary in all pools on many different time scales including interannually, seasonally, and daily. This variation might slow or prevent adaptation to temperature. Indeed, the evolution of other species has been affected by temperature variation. For example, wing melanin, a key thermoregulatory trait in the alpine butterfly *Colias meadii*, has evolved more slowly than expected in response to recent climate change due to temporal variation in selection (Kingsolver & Buckley, 2015). Also, Bonebrake and Deutsch (2012) demonstrated that in temperate regions like our study area, the effect of seasonal temperature variation swamps the effect of spatial temperature variation on the adaptation of species temperature tolerances.

A large body of research suggests that adaptation to temperature variation at broad-spatial scales results in geographic differences in the evolutionary and plastic potential of species (Nadeau et al., 2017). This geographic pattern is especially evident when comparing species from tropical and temperate locations. However, the question remains if temperature variation regularly affects the sensitivity and response capacity of species at finer-spatial scales. *Daphnia* is well-known for differentiating across fine spatial scales (Declerck et al., 2001), providing the basis for our original predictions. Here, we did not observe the predicted relationships, likely due to high gene flow, and variable selection. However, other studies have observed fine-scaled differences in a variety of traits among populations from environments with different amounts of temperature variation, despite the potential for gene flow among populations (Brahim et al., 2019; Freidenburg & Skelly, 2004; Johansson et al., 2016; Richter‐Boix et al., 2015; Skelly, 2004; Skelly & Freidenburg, 2000). Thus, our work provides a cautionary tale in extrapolating evidence for fine-scaled adaptation across sites for the same species. A more detailed understanding of the spatial and temporal scale of natural selection, existing genetic variation, gene flow, and fitness tradeoffs will likely be necessary to predict local adaptation to climate change across species ranges. We suspect microgeographic adaptation to temperature variation will be more common in systems (a) with large differences in temperature variation (e.g., thermal springs versus surrounding lake water; Johansson et al., 2016) or (b) strong selection (e.g., selection on growth rate in vernal pools; Skelly, 2004). More studies are needed to determine the scales at which differences in temperature variation are likely to affect evolutionary and plastic potential and what ecological factors affect those scales. Such studies could significantly improve our predictions of which species will be most vulnerable to climate change, where they will be vulnerable, and help guide conservation strategies to minimize biodiversity loss in a changing climate.

## Supplementary Material

qrad042_suppl_Supplementary_Tables_S2_Datas_S3

## Data Availability

The data and code that support the findings of this study are openly available on the Dryad Digital Repository at https://datadryad.org/stash/dataset/doi:10.5061/dryad.s7h44j1cv.
