# Supplementary material for: Macroecological predictors of evolutionary and plastic potential do not apply at microgeographic scales for a freshwater cladoceran under climate change
Source: Evol Lett. 2023 Oct 12;8(1):43–55. doi: 10.1093/evlett/qrad042 (PMC10872021; doi:10.1093/evlett/qrad042)
Supplement: qrad042_suppl_Supplementary_Tables_S2_Datas_S3 [file qrad042_suppl_supplementary_tables_s2_datas_s3.pdf]

Macroecological Predictors of Evolutionary and Plastic Potential Do Not Apply at Microgeographic Scales for a Freshwater Cladoceran Under Climate Change

Supplemental Material

Christopher P. Nadeau, and Mark C. Urban.

Supplemental Material S1 – Annual Temperature Summaries

Table S1. Summaries of temperature for the 10 focal freshwater rock pools in 2016, 2017, and 2018. Note, temperature data was not available for all years. Maximum refers to the average daily maximum temperature during the hottest month (August). Mean referes to the average hourly temperature between Jun3 15 and October 15. SD refers to the daily standard deviation in maximum temperature recorded between June 15 and October 15. Autocorrelation refers to the predictability of maximum daily temperature recorded between June 15 and October 15 measured as the range of a variogram model (higher autocorrelation equals higher predictability). Seasonal range is the range daily maximum temperature between June 15 and October 15.

	Maximum			Mean			SD			Autocorrelation			Seasonal Range		
Pool	2016	2017	2018	2016	2017	2018	2016	2017	2018	2016	2017	2018	2016	2017	2018
1	23.0		23.7	19.6		19.6	1.1		1.1	1.33		1.26	12.0		13.8
2	24.2	22.7	24.7	19.3	18.6	19.4	1.8	1.9	1.9	1.11	1.19	0.92	15.1	11.6	18.0
3	25.0	23.8	25.4	19.8	19.0	19.6	1.7	1.9	1.7	1.17	1.08	0.99	16.8	12.9	18.2
4		24.3	25.7		19.3	20.1		2.1	1.8		1.07	0.98		13.3	18.8
5		23.3	26.1		19.1	19.8		1.7	2.1		1.16	0.91		11.2	18.9
6	25.5	24.7	26.2	19.8	19.2	19.8	1.8	2.1	2.0	1.10	1.00	0.90	15.9	12.3	19.6
7		24.4	26.8		19.3	20.3		2.3	2.1		1.02	0.85		13.0	19.3
8		25.6	27.0		18.8	19.7		3.3	2.7		0.88	0.90		17.7	21.1
9	27.5	26.8	28.0	20.4	19.5	20.4	2.5	2.9	2.6	0.96	0.89	0.83	17.4	16.8	20.9
10			28.1			20.4			2.8			0.83			22.6

Supplemental Material S2 – Sample Sizes by Pool and Year

Table S2: The number of clones and individuals per clone used to estimate *D. magna* fitness and CTmax in each pool and year.

	Fitness								Ctmax							
	Number of Clones				Number of Individuals				Number of Clones				Number of Individuals			
	2017		2018		2017		2018		2017		2018		2017		2018	
**Pool**	**20°C**	**25°C**	**20°C**	**25°C**	**20°C**	**25°C**	**20°C**	**25°C**	**20°C**	**25°C**	**20°C**	**25°C**	**20°C**	**25°C**	**20°C**	**25°C**
1	0	0	14	14	0	0	32	36	0	0	14	14	0	0	31	36
2	1	1	22	22	3	1	52	50	1	1	21	22	3	1	49	48
3	0	0	14	14	0	0	39	37	0	0	14	14	0	0	38	32
4	7	7	0	0	20	10	0	0	7	7	0	0	18	11	0	0
5	1	1	7	7	1	2	17	18	1	1	7	7	1	2	17	17
6	2	1	7	7	2	1	15	15	2	1	7	7	2	1	15	15
7	5	4	6	6	9	6	12	14	6	4	5	6	10	5	11	14
8	6	4	0	0	13	4	0	0	6	4	0	0	13	4	0	0
9	0	0	18	18	0	0	43	43	0	0	18	19	0	0	44	44
10	0	0	17	18	0	0	45	40	0	0	17	17	0	0	44	36

Supplemental Material S3 – Model Descriptions

Definitions:
y: CTmax or fitness (depending on analysis)
i: individual
t: experimental trail (only used when analyzing CTmax)
c: clone
p: pool of origin
e: environmental
σ: variance
α: random intercept
β: coefficient (β_0 is the intercept)
int: interaction
year: year individual *i* was collected (reference is 2017)
pool temp: average maximum temperature in pool *p*
exp. temp: lab developmental temperature (reference is 20°C) for individual *i*
SD: daily temperature variation in pool *p*
pred: daily temperature predictability in pool *p*

Microgeographic Adaptation and Fluctuating Selection Hypotheses (including only data from Daphnia raised at 20°C):

Model M1-0 (no genetic variation)

$$y_{i,t} \sim Normal(\mu_{i,t}, \sigma_e)$$
$$\mu_{i,t} = \beta_0 + \beta_{year} * year_i + \alpha_t$$
$$\alpha_t \sim Normal(0, \sigma_t)$$

Model M1-1 (genetic variation, where $\boldsymbol{\sigma_c}$ is an estimate of the genetic variance)

$$y_{i,t,c} \sim Normal(\mu_{i,t,c}, \sigma_e)$$
$$\mu_{i,t,c} = \beta_0 + \beta_{year} * year_i + \alpha_t + \alpha_c$$
$$\alpha_t \sim Normal(0, \sigma_t)$$
$$\alpha_c \sim Normal(0, \sigma_c)$$

Model M1-2 (CTmax or fitness differs among pools)

$$y_{i,t,c,p} \sim Normal(\mu_{i,t,c,p}, \sigma_e)$$
$$\mu_{i,t,c,p} = \beta_0 + \beta_{year} * year_i + \alpha_t + \alpha_c + \alpha_p$$
$$\alpha_t \sim Normal(0, \sigma_t)$$
$$\alpha_c \sim Normal(0, \sigma_c)$$
$$\alpha_p \sim Normal(0, \sigma_p)$$

Model M1-3 (microgeographic adaptation)

$$y_{i,t,c,p} \sim Normal(\mu_{i,t,c,p}, \sigma_e)$$
$$\mu_{i,t,c,p} = \beta_0 + \beta_{year} * year_i + \beta_{pool\ temp} * pool\ temp_p + \alpha_t + \alpha_c + \alpha_p$$
$$\alpha_t \sim Normal(0, \sigma_t)$$

$$\alpha_c \sim Normal(0, \sigma_c)$$
$$\alpha_p \sim Normal(0, \sigma_p)$$

Model M1-4 (fluctuating selection, where the $\boldsymbol{\sigma_c}$ is estimated separately for each pool)

$$y_{i,t,c,p} \sim Normal(\mu_{i,t,c,p}, \sigma_e)$$
$$\mu_{i,t,c,p} = \beta_0 + \beta_{year} * year_i + \alpha_t + \alpha_c$$
$$\alpha_t \sim Normal(0, \sigma_t)$$
$$\alpha_c \sim Normal(0, \sigma_{c,p})$$

Adaptive Plasticity Hypotheses (including data from Daphnia raised at 20°C and 25°C):

Model M2-0 (no plasticity)

$$y_{i,t,c,p} \sim Normal(\mu_{i,t,c,p}, \sigma_e)$$
$$\mu_{i,t,c,p} = \beta_0 + \beta_{year} * year_i + \alpha_t + \alpha_c + \alpha_p$$
$$\alpha_t \sim Normal(0, \sigma_t)$$
$$\alpha_c \sim Normal(0, \sigma_c)$$
$$\alpha_p \sim Normal(0, \sigma_p)$$

Model M2-1 (plasticity, where $\beta_{exp.temp}$ is an estimate of plasticity)

$$y_{i,t,c,p} \sim Normal(\mu_{i,t,c,p}, \sigma_e)$$
$$\mu_{i,t,c,p} = \beta_0 + \beta_{year} * year_i + \beta_{exp.temp} * exp.temp_i + \alpha_t + \alpha_c + \alpha_p$$
$$\alpha_t \sim Normal(0, \sigma_t)$$
$$\alpha_c \sim Normal(0, \sigma_c)$$
$$\alpha_p \sim Normal(0, \sigma_p)$$

Model M2-2 (adaptive plasticity associated with temperature variation)

$$y_{i,t,c,p} \sim Normal(\mu_{i,t,c,p}, \sigma_e)$$
$$\mu_{i,t,c,p} = \beta_0 + \beta_{year} * year_i + \beta_{expTemp} * exp.temp_i + \beta_{SD} * SD_p + \beta_{int} * exp.temp_i * SD_p + \alpha_t + \alpha_c + \alpha_p$$
$$\alpha_t \sim Normal(0, \sigma_t)$$
$$\alpha_c \sim Normal(0, \sigma_c)$$
$$\alpha_p \sim Normal(0, \sigma_p)$$

Model M2-3 (adaptive plasticity associated with temperature predictability)

$$y_{i,t,c,p} \sim Normal(\mu_{i,t,c,p}, \sigma_e)$$
$$\mu_{i,t,c,p} = \beta_0 + \beta_{year} * year_i + \beta_{exp.temp} * exp.temp_i + \beta_{pred} * Pred_p + \beta_{int} * exp.temp_i * Pred_p + \alpha_t + \alpha_c + \alpha_p$$
$$\alpha_t \sim Normal(0, \sigma_t)$$
$$\alpha_c \sim Normal(0, \sigma_c)$$
$$\alpha_p \sim Normal(0, \sigma_p)$$

Temperature Effects on Heritability:

Model M3-0 (temperature does not affect genetic or not-genetic variance)

$$y_{i,t,c} \sim Normal(\mu_{i,t,c}, \sigma_e)$$
$$\mu_{i,t,c} = \beta_{0,exp.temp} + \beta_{year} * year_i + \alpha_t + \alpha_c$$
$$\alpha_t \sim Normal(0, \sigma_t)$$
$$\alpha_c \sim Normal(0, \sigma_c)$$

Model M3-1 (temperature affects not-genetic variance, i.e., $\boldsymbol{\sigma_e}$ differs for each experimental temperature)

$$y_{i,t,c} \sim Normal(\mu_{i,t,c}, \sigma_{e,exp.temp})$$
$$\mu_{i,t,c} = \beta_{0,exp.temp} + \beta_{year} * year_i + \alpha_t + \alpha_c$$
$$\alpha_t \sim Normal(0, \sigma_t)$$
$$\alpha_c \sim Normal(0, \sigma_c)$$

Model M3-2 (temperature affects genetic, i.e., $\boldsymbol{\sigma_c}$ differs for each experimental temperature)

$$y_{i,t,c} \sim Normal(\mu_{i,t,c}, \sigma_e)$$
$$\mu_{i,t,c} = \beta_{0,exp.temp} + \beta_{year} * year_i + \alpha_t + \alpha_c$$
$$\alpha_t \sim Normal(0, \sigma_t)$$
$$\alpha_c \sim Normal(0, \sigma_{c,exp.temp})$$

Model M3-3 (temperature affects genetic and non-genetic variance)

$$y_{i,t,c} \sim Normal(\mu_{i,t,c}, \sigma_{e,exp.temp})$$
$$\mu_{i,t,c} = \beta_{0,exp.temp} + \beta_{year} * year_i + \alpha_t + \alpha_c$$
$$\alpha_t \sim Normal(0, \sigma_t)$$
$$\alpha_c \sim Normal(0, \sigma_{c,exp.temp})$$

Supplemental Material S4 – Simulations to Evaluate Detectable Effect Size

Models suggesting CTmax was associated with pool temperature (M1-3), CTmax plasticity was associated with temperature variation and predictability (M2-2 and M2-3), and fitness plasticity was associated with temperature predictability (M2-3) received some support. However, the 95% CI of the important coefficients overlapped zero, suggesting no support for the hypothesis. Credible intervals that overlap zero could indicate a lack of power to detect biologically meaningful effects. Hence, we used a series of simulations to evaluate whether we had the power to detect biologically meaningful effects for model M1-3 with CTmax as the response, model M2-2 with CTmax as the response, and model M2-3 with fitness as the response. First, we simulated new response data (i.e., CTmax or fitness) using the model of interest (Supplemental Data S4) and the observed predictor data. When simulating the data, we specified an effect size for the coefficient of interest (i.e., the pool temperature coefficient in model M1-1 and the interaction coefficient in M2-2 and M2-3) and we used the mean of the posterior from the fitted model for all other coefficients and variance terms. The simulated response data differed from the observed data due to randomly generated errors and random intercept terms as specified in the model (Supplemental Data S4). We then refit the model to the simulated data and extracted the 95% credible interval for the parameter of interest. We repeated the simulation 100 times and calculated the proportion of simulations where the 95% credible interval did not overlap zero. We assumed we had power to detect the specified effect size if 80% of the simulations had 95% credible interval that did not overlap zero. We repeated the analysis with the parameters of interest set to the following values: 0.0 - 0.3 (interval 0.1; Fig. S4-1A) for the pool temperature coefficient in model M1-1 with CTmax as the response variable; 0.00 – 0.35 (interval 0.05; Fig. S4-1B) for the interaction coefficient in model M2-2 with CTmax as the response variable; and 0.00 – 0.10 (interval 0.01; Fig. S4-1C) for the interaction coefficient in model M2-2 with fitness as the response variable. The simulations suggested that we had the power to detect a 0.2°C increase in CTmax for every 1°C increase in pool temperature, and 0.20°C increase in CTmax plasticity for every 1°C in the standard deviation of pool temperature, and an 0.06 increase in fitness for every unit of predictability in pool temperature (Fig. S4-1). These results suggest that we had sufficient power to detect biologically relevant effects in all three situations.

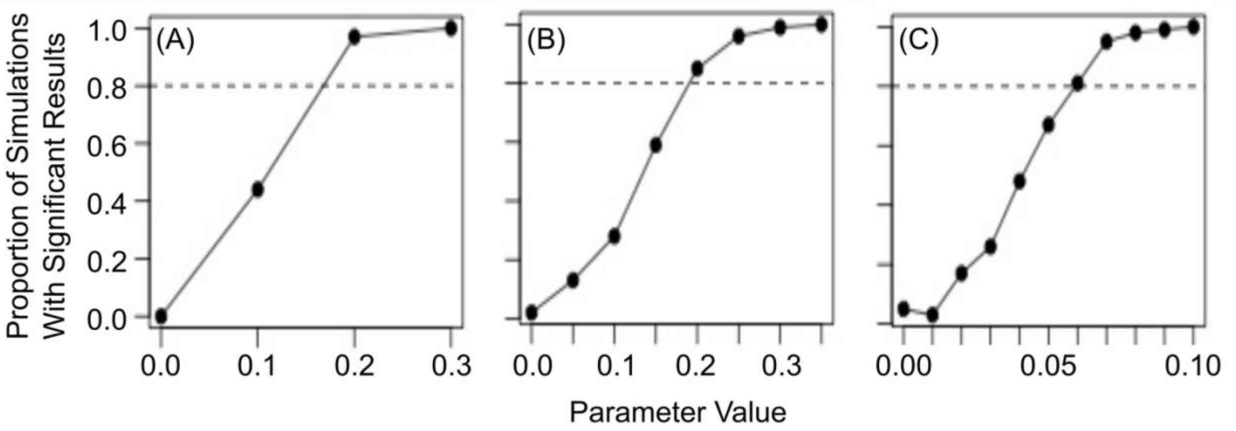

Figure S4-1. The proportion of 100 simulations where the 95% credible interval for the parameter of interest did not overall zero (i.e., was significant) for: (A) the relationship between CTmax and the maximum temperature in the pool of origin from model M1-1(i.e., the microgeographic adaptation hypothesis); (B) the interactive effect of developmental temperature and temperature variation on CTmax from model M2-2 (i.e., the adaptive plasticity hypothesis); and the interactive effect of developmental temperature and temperature predictability on fitness from model M2-3 (i.e., the adaptive plasticity hypothesis). The parameter values are: (A) the pool temperature coefficient and (B and C) the interaction coefficient. We assumed we had power to detect the specified effect size if 80% of the simulations (dashed line) had 95% credible interval that did not overlap zero.

Supplemental Material S5 – Simulations to Evaluate How Sample Size Affects Estimates of Genetic Variance

We conducted simulations to evaluate (1) how the number of individuals per clone and (2) how the within-clone variance affects estimates of genetic variation.

To address the first point, we simulated a sample with 15 clones from a single pool with an average CTmax of 37°C. We set the variance among clones (i.e., the genetic variance) to 0.05 and the variance within clones (i.e., environmental variance plus sampling error) to 0.17, which are similar to the estimates from our results (see Fig. 3B). We then simulated 5 data sets, by creating a sample with either 1.7 (i.e., 1/3 of clones are represented by 1 individual and all other clones have 2 individuals), 2, 3, 4, or 5 individuals per clone. Last, we fit a mixed effect model to each data set using the lme4 package in R to estimate the genetic variance. We did not use a Bayesian model, as we did in the paper, to reduce computation time, but this should make little difference. We repeated the simulation 1000 times and plotted a box plot of genetic variances (Figure S5-1).

The results demonstrate the estimates of genetic variance are unbiased (i.e., the median is very close to the true genetic variance). Of course, the precision is affected by the number of individuals per clone, but with only 2 clones the estimates are fairly precise and increasing the number of individuals per clone only increases the precision slightly.

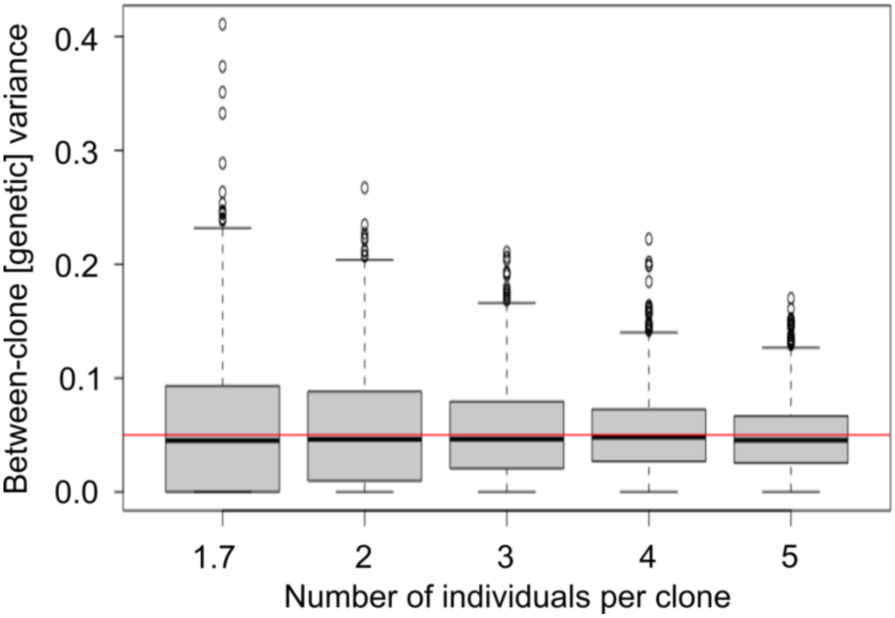

Figure S5-1. Box plots of 1000 estimates of genetic variance from mixed effects models using simulated data with different numbers of individuals per clone. The horizontal red line is the true genetic variance in the simulations.

To address the second point, we simulated a sample with 15 clones from a single pool with an average CTmax of 37°C and 2 individuals per clone. We set the variance among the clones to

0.05. We created 5 data sets with different levels of within clone variance, including: 0.05, 0.10, 0.15, 0.20, 0.25. We then fit a mixed model using the lme4 package in R to estimate the genetic variance. We repeated the simulation 1000 times and plotted a box plot of genetic variances (Figure S5-2).

The results demonstrate that the estimates of genetic variance are unbiased regardless of the within-clone variation. Moreover, the estimates are fairly precise, especially with the amount of within-clone variance (~0.15) we observed in our study.

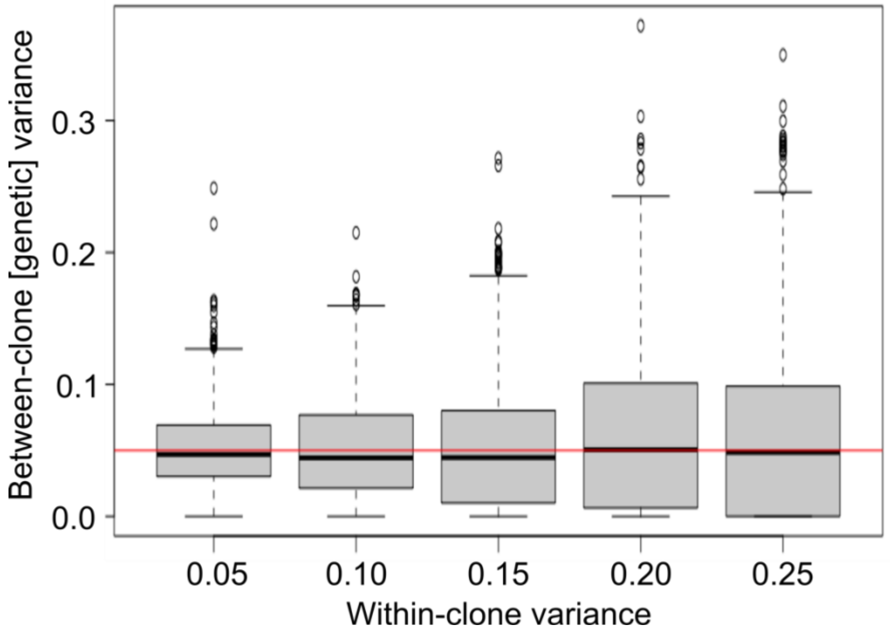

Figure S5-2. Box plots of 1000 estimates of genetic variance from mixed effects models using simulated data with different within-clone variances. The horizontal red line is the true genetic variance in the simulations.
